# The effects and underlying mechanism of extracorporeal shockwave therapy on fracture healing

**DOI:** 10.3389/fendo.2023.1188297

**Published:** 2023-05-24

**Authors:** Fuxian Lv, Zhenlan Li, Yuling Jing, Liyuan Sun, Zhiwei Li, Haoyang Duan

**Affiliations:** Department of Rehabilitation Medicine, First Hospital of Jilin University, Chang chun, China

**Keywords:** extracorporeal shockwave therapy, fracture, osteogenesis, growth factor, review

## Abstract

The clinical efficacy of ESWT in treating bone non union has been widely recognized, but the biological mechanism of ESWT promoting bone non union healing is still unclear. ESWT can make old callus micro fracture through mechanical conduction, form subperiosteal hematoma, promote the release of bioactive factors, reactivate the fracture healing mechanism, rebalance the activities of osteoblasts and osteoclast, promote the angiogenesis of fracture site, and accelerate the healing of bone nonunion.Over recent years, great efforts have been made by both scientists and clinicians to explore the underlying mechanism behind the healing effect of ESWT on bone fractures. In this review, we introduced the growth factors during osteogenesis induced by ESWT hoping to provide new insights in the clinical use of ESWT.

## 1 Introduction

Researchers from various medical centers around the world have expounded on the definitions of delayed union and non union of fractures based on their own understanding, but currently there is no definitive unified understanding of the definitions of delayed union and non union of fractures. Human fractures usually reach bone healing within about 3 months, but there are still some patients who cannot achieve healing within normal time due to various factors, such as biological factors, technical factors, or other factors.

In the past few decades of research, it has been found that fracture healing is a unique process leading to bone regeneration, which has led to in-depth research on this complex process, with a focus on the role of cytokines and growth factors in fracture healing. So far, researchers have found that more than 50 cytokines play an important role in fracture healing, such as bone morphogenetic protein (BMP), transforming growth factor-β(TGF-ß), binding factor-α1 (CBF-α1), vascular endothelial growth factor (VEGF), insulin-like growth factor (IGF), fibroblast growth factor (FGF), and platelet-derived growth factor (PDGF) (1, 2).

Extracorporeal shockwave (ESW) is a special form of mechanical waves that rapidly release over a period of a very short time. The energy of ESW is about 1000 times higher compared to ultrasound energy. In the 1980s, ESW was successfully applied to treat urolithiasis. After continuous exploration of ESW, Haupt was first to discover that ESW can activate osteoblasts and promote osteogenesis.ESW of the appropriate energy applied to the human body can lead to tiny fresh fractures and microvascular regeneration, and can also stimulate the release of growth factors, which ultimately contribute to tissue regeneration and fracture healing. Being a novel and non-invasive treatment, ESWT is currently considered as an effective strategy to promote healing quality in patients with delayed and nonunion fractures (3–5). ESWT has many advantages over traditional surgical treatments due to its noninvasiveness, safety and cost-effectiveness (6, 7).

ESWT, as a non-invasive treatment method for stimulating osteogenesis, has been proven to activate cytokines related to bone formation such as BMP. At the same time, ESWT can significantly promote the expression of TGF - ß, thereby promoting the growth and differentiation of bone marrow stromal cells into osteoprogenitor cells, thereby accelerating bone regeneration. ESWT can also promote the early expression of growth factors related to angiogenesis, including endothelial nitric oxide synthase and nitric oxide, VEGF, and proliferating cell nuclear antibody, thereby inducing angiogenesis, cell proliferation, and tissue regeneration and repair. At present, there is no consensus on the biological mechanism of ESWT promoting fracture healing. This article reviews the research on the biological effects of ESWT and the biological mechanism of ESWT promoting fracture healing.

## 2 Biological effects of ESW

### 2.1 Biological effects of stress

ESW is propagated in tissues of different densities, resulting in stress gradients caused by differences in the acoustical and mechanical properties of the tissue. Studies have shown that when ESW penetrate the human body, different mechanical stress effects (compressive stress and tensile stress gradient) may occur at the interface of different tissues due to the characteristics of different tissues, such as soft tissues (like fat, tendons and ligaments) and skeletal tissues, stones, calcifications and similar (8). Shear force is generated inside the object, leading to different tensile and compressive stresses on the cells (9). Tensile stress can cause detachment between tissues and can promote microcirculation, compressive stress can cause elastic deformation in cells and increase cells oxygen for therapeutic purposes. Of course, this may also be one of the mechanisms through which ESW induces tissue damage.

### 2.2 Biological effects of cavitation

Cavitation generally refers to the process of bubble generation, growth and collapse in liquids. When there is an empty bubble flowing in the liquid, surrounding pressure increases suddenly, and the volume of empty bubble dramatically reduces or collapses. Since the bubble collapse process occurs in an instant (microseconds), it produces locally very high instantaneous pressure. When the collapse occurs near the solid surface, the extremely high pressure generated by the constantly collapsing empty bubble in the fluid destroys the solid surface. Cavitation effect refers to the bubble formation process and its active role triggered by thermal, acoustic or mechanical mechanisms in liquid (10). The bubble is formed by the liquid burst under the action of acoustic tension. The tension produced by ESW can cause the local body tissue to produce low pressure and generate a large number of tiny bubbles. The bubbles rapidly inflate and rupture under the action of tension wave, resulting in high-speed liquid micro-jetting and impact effect. Subsequently, this cavitation effect at the appropriate dose is conducive to increasing the permeability of the cell membrane, unblock the isolated capillaries, and to loosen the adhesion of soft tissue joints (11, 12).

### 2.3 Biological effects of high density tissue lysis

The most common clinical disease with high-density tissue is urinary calculus, for which ESW has been widely used (13, 14). The basic principle of ESW cracking high density tissue is stress. ESW enters from the front surface of high-density tissue and impacts on its surface. If its energy and pressure exceed the compressive strength limit, cracks are formed on the front surface of high-density tissue. After ESW enters the front surface of high-density tissue, a strong squeeze and stretch appear inside, and high-density tissue surface cracks continue to expand and produce more cracks in its interior. When the shock wave passes through the high-density tissue to reach the back interface, a portion is reflected and re-applied to the high-density tissue while the remaining portion passes through the high-density tissue to the soft tissue, causing tension to break the high-density tissue rear surface. Because of the heterogeneity of high-density tissue internal structure, the formation of complex stress fields by pressure and tension causes the high-density tissue to rupture from the outside to the inside, resulting in multiple ruptures at multiple interfaces.

### 2.4 Metabolic activation effect

Metabolic activation effects are directly caused by mechanical effects. ESW can activate both intra- and extra-cellular ion exchange processes, and accelerate the removal and uptake of end products of metabolism (15, 16). Neovascularization is beneficial for activating metabolic effects in the body, and VEGF is an important factor in angiogenesis. ESW can activate monomer G protein signal pathway, make receptor tyrosine kinase inhibit signal molecules of extracellular signal related kinase and cytoplasmic protein kinase of overpressure, and induce hypoxia inducible factor-1 α Binding to the promoter of VEGF, thereby increasing VEGF-1 α The gene expression of ESWT generates more VEGF, which elucidates how ESWT activates metabolic effects in the body (17). ESWT can also promote angiogenesis and improve tissue blood supply by regulating endothelial nitric oxide synthase and increasing the expression of platelet endothelial cell adhesion molecules, thus activating the metabolic effect of the body (9). In addition, the expression of vascular endothelial growth factor and nitric oxide synthase significantly increased after ESW intervention, which can increase the number of blood vessels at the site of action, facilitate the transportation of nutrients and maintain cell metabolism, thereby promoting the activation of metabolic effects in the body (18).

### 2.5 Osteogenesis effect

ESW have an impact on bone tissue, Produce minor damage in the treated bone (focal point) without damaging adjacent soft tissues. The osteogenic promoting effect of extracorporeal shock waves occurs at the interface between the cortical and reticular parts of the bone, and these treatments trigger micro injuries to stimulate and reactivate the bone’s healing ability in non healing fractures (19). ESWT can cause microfractures in old callus through mechanical conduction, forming subperiosteal hematoma, promoting the release of bioactive factors, inducing the expression of various angiogenic and osteogenic growth factors, significantly increasing the number of new blood vessels in the tissue, and thus accelerating the healing of bone non union (20). The osteoblasts convert mechanical signals into biochemical signals to osteoblasts and osteoclasts, which in turn affect the reconstruction of bone tissue. The ESW of appropriate intensity can agglutinate and activate osteoblasts, thus promoting osteogenesis and fracture healing.

### 2.6 Time dependence and cumulative effect

Wang et al. used ESWT to treat fractures in the right tibia in dogs with energy flow density of 0.18mJ/mm^2^, where the left tibia without ESWT was selected as the control. Imaging checks were performed at 1,4,8 and 12 weeks post- treatment. Briefly, no significant difference in the formation of poroma was observed between the two groups 8 weeks after treatment, while higher poroma formation was found in the treatment group at 12 weeks post- treatment. In addition, histological observation confirmed that the cortex in treatment group appeared thicker and dense compared to the control group, suggesting that the treatment with ESWT had “time-dependent” quality (21). Furthermore, Maier et al. have shown that the longer ESWT led to the better treatment effect in chronic rotator cuff calcification tendonitis (22). Moreover, Chen and his team have performed a prospective study of ESWT treatment of heel pain. Their results suggested that 12 weeks after the first treatment, 20.6% of patients experienced complete pain relief, 52.90% experienced near full relief, 17.7% experienced partial relief. 24 weeks after treatment, some patients reported further improvement, thus confirming a time-dependent quality of this approach. Interestingly, those who did not respond to initial treatment, showed some relief after the second treatment, thus suggesting a potential cumulative effect of ESW (23).

## 3 Impact of ESW on fracture repair

Osteopromotive growth factors have a key role in enhancing bone formation at different stages of the fracture repair process. Studies have shown that ESW influences the biological activity of osteoblasts, and the expression levels of certain cytokines (24, 25). Regarding the biological effects of ESW, many researchers have conducted extensive research in the fields of osteogenic growth factors, including BMP, TGF-ß, CBF-α1, VEGF, IGF, FGF, PDGF.

### 3.1 BMP

BMP is a hydrophobic acid polypeptide which can induce the proliferation and differentiation of primary stromal cells to form bone and chondrocytes (26, 27). Bilem et al. have confirmed that BMP can induce ossification (28). ESW can increase the levels of BMP, which in turn can induce the osteogenic differentiation of mesenchymal stem cells during new bone formation (29). Among 20 BMPs, BMP-2 has the most prominent ability to induce ossification. At the same time, BMP-2 can significantly promote bone formation and remodeling (30). Huang and colleagues have used sixty rats as a model for double proximal tibial osteotomy and inner fixation. The osteotomy site in the left tibia was treated with roughly focused ESWT once at an energy density of 0.26 mJ/mm^2^, 60 doses/min, and 2000 pact quantities. The contralateral right tibia was left untreated and was used as a control. The obtained results revealed that ESWT may promote the expression of BMP-2 in the fracture area in rats, that BMP-2 may act synergistically and that it may lead to a significant enhancement of bone formation and remodeling (31). Furthermore, Chert et al. have shown that the ideal energy density of ESW can increase the number of callus and bone density, as well as to promote bone formation; in the process of compact bone formation, BMP expression also increases. Accordingly, BMP has an important role in the promotion of osteoblast proliferation and bone regeneration by ESWT (32).

### 3.2 TGF-ß

TGF-ß is a kind of polypeptide with many functions. It is widely expressed in human cells and is mostly synthesized by osteoblasts and osteoclasts. It is most abundantly present in bone tissue, where its concentrations are much higher compared to other tissues. TGF-ß has different roles in the regulation of osteoblast-secreting cytokines, extracellular matrix production and cell maturation (33). TGF-ß exerts its biological effects mainly by inducing the transcription of some target genes. Most scholars believe that TGF-ß gets accumulated in the process of os cartilaginea before osteogenesis, and then regulates the expression of alkaline phosphatase (AKP), collagen II (Col-II) and other tissue-specific proteins (34). *In vitro* studies have verified that Rat bone marrow stromal cells respond to ESWT by increasing TGF-ß1 gene expression, thus causing cell proliferation and osteoblastic differentiation. Chen et al. have treated the defect site of the femoral shaft with ESW in 0.16 mJ/mm ^2^, 2500 times. Morphological changes in the bone tissue and in cells of the defect area were observed at different stages, and the expression of TGF-β was detected. After 3 days of ESW treatment, the expression TGF-β significantly increased (P <0.05) (23). Accordingly, TGF-ß not only regulates the proliferation and differentiation of cells, but also acts as a strong chemotactic factor for osteoblasts, which can move and aggregate osteoblasts from low concentrations of TGF-ß to high concentrations. Wang and colleagues have observed the proliferation and differentiation of rat femur fracture site after a treatment with ESW in 0.16 mJ/mm 2, 2, 500 times, and have found that ESW promotes the colony formation of pre-osteoblast and stromal cells (35). Thus, in the process of promoting osteogenesis by ESW, TGF-β may be relevant for chemotaxis and may accelerate the mitosis of bone marrow stromal cells.

### 3.3 CBF-α1

CBF-α1 is a master gene in the process of osteoblast differentiation, closely related to osteoblasts, chondrocytes and osteoclasts, with a crucial role in bone cell maturation and angiogenesis. It is an indispensable activator of osteoblast differentiation (36, 37). Mice that are CBF-α1-deficient, lack the necessary osteoblasts and thus fail to form bone tissue (38). CBF-α1 is also the core factor in osteoblast differentiation and function regulation, which is the earliest emergence and most specific marker in osteoblast differentiation. It has an important role in the regulation of osteocalcin gene expression in mature osteoblasts. Masuhiro et al. have found that CBF-α1 is a master gene in osteoblast differentiation and bone formation. The deletion of this gene leads to bone dysplasia or termination, and at the same time CBF-α1 can regulate the transcription of the target gene, inducing the expression of bone-specific proteins (39). ESWT may regulate the transcriptional function of CBF-α1 through the MAPKs pathway, which not only enhance the expression of CBF-α1 but also enhance the binding of CBF-α1 to DNA. Wang et al. have found that ESWT can increase ERK activity and promote osteoblastic differentiation; superoxidase inhibits phosphorylation of ERK which in turn leads to decreased expression of the specific osteogenic transcription factor CBF-α1. ESW can activate the MAPK pathway, which leads to the activation of CBF-α1, and promotes osteoblast growth and maturation (40).

### 3.4 VEGF

VEGF promotes vascular endothelial cell mitosis and can stimulate the proliferation of vascular endothelial cells, enhance hemodialysis, mediate vascular endothelial cell migration and thus promote angiogenesis. VEGF is considered to be the most potent and specific angiogenic inducible factor (41, 42). One of the mechanisms underlying nonunion treatment is promotion of vascularization, which highlights the importance of sufficient vascular network during osteogenesis. If VEGF secretion is insufficient, vascularization process gets inhibited, inflammatory response to the process of cell proliferation blocked, and the process of fracture healing extended or even stagnated (43). Lv et al. have suggested that VEGF has the dual role in promoting angiogenesis and osteogenesis, which can be expressed in the fracture of the inner bone adventitia lining of mesenchymal cells, chondrocytes and trabecular bone around the osteoblasts, where VEGF combined with alkali fibroblast growth factor can stimulate blood vessel formation and supply blood thus promoting osteogenesis, and simultaneously promoting the calcification of cartilage and remodeling of bone tissue (44, 45). Moreover, Chen and colleagues have found that ESW can promote the expression of VEGF in mesenchymal cells of defected femur rats (23). Using NADPH oxidase blockade, Wang et al. have confirmed that the secretion of VEGF in osteoblasts increases after ESWT intervention, and that ESWT can induce the expression of VEGF, which is of great significance for early bone formation and remodeling (46).

### 3.5 IGF

IGF has a medium - promoting mitotic effect on osteoblasts, it regulates cell cycle G1 activity, and has insulin-like effects. IGF is an important growth factor secreted by bone cells which regulates osteoblast activity in an autocrine and paracrine manner (47, 48). IGF is an important factor regulating the function and metabolism of bone cells. It can reduce the degradation of collagen, increase the deposition of bone and promote the differentiation and maturation of osteoblasts. It can also promote the proliferation, differentiation and recruitment of osteoblasts (49). The initial step in fracture repair is the osteoblast recruitment in repair section. Preclinical investigation have shown that IGF promotes osteoblast recruitment in a dose-dependent manner, and in the early bone repair process, it promotes the recruitment of osteoblasts to the repair site, thus revealing a very important role (50, 51). In the osteoblast culture model, McCarthy et al. have found that the osteoblast-synthesizing protein acts *via* the IGF receptor pathway and that the ligand binds to the IGF receptor which is determined by a specific intracellular pathway of the osteocytes. The rapid change in the number of the IGF receptor phenotypes appeared to be at least partially effective after protein translation, which can help explain the autocrine or paracrine pathways of IGFs in bone tissue (52). Moreover, Chen et al. have suggested that ESWT alone in mouse model led to intensive cell proliferation, neovascularization and tissue regeneration at the site of injury. At the early stages of repair, cell proliferation is directly proportional to IGF expression, and the number of IGFs continues to increase during treatment. This suggests that physical ESW can increase the mitotic response of tissue cells and promote tissue repair (53).

### 3.6 FGF

FGF is a pleiotropic growth factor, which in combination with receptor (fibroblast growth factor receptors, FGFR) regulates the cell proliferation, migration, differentiation and apoptosis (54, 55). FGF/FGFR have an important role in both embryonic bone formation and post-natal skeletal development. FGF/FGFR signaling mainly regulate bone formation by regulating the expression of various genes involved in bone progenitor cell replication, osteoblast differentiation and apoptosis (56). In the process of osteoblast differentiation, FGF/FGFR signaling has both positive and negative regulatory roles. In addition to its own role, FGF signaling can also interact with BMP signaling to form a complex regulatory relationship which is involved in regulating osteoblast differentiation and affecting the expression of various genes at different stages of osteoblast differentiation (57). It has been reported that FGF levels in the body significantly increase (p <0.05) 24 hours after ESW treatment and significantly correlate with the number of pulses (p <0.05), thus promoting cell proliferation and differentiation. By inducing FGF synthesis, ESWT can promote bone healing (58).

### 3.7 PDGF

PDGF is an important polypeptide growth factor produced by a variety of cells. It is a mitogenic stimulator for many kinds of cells, and it has chemotactic effect on mesenchymal cells, osteoblasts and osteoblasts. It is also one of the strongest bone growth factors (59). PDGF can both promote the formation of bone tissue by osteoblasts and stimulate bone resorption playing a two-way role in the regulation of bone remodeling. While PDGF can promote the synthesis of osteoblast DNA, cells replication, the synthesis of collagen and non-collagen, the formation of new blood vessels is one of the key factors in the successful repair of trauma (60). PDGF is one of the most important wound healing factors in the body. Over recent years, numerous experiments have shown that PDGF can promote the damage repair of bone, cartilage and tendon tissue (61). Studies have shown that low-intensity physical stimulation such as pulsed ultrasound can increase PDGF secretion, thereby promoting mesenchymal cell proliferation and migration (62).

## 4 Summary

Currently the use of ESWT for treating fractures, especially the ones with delayed union and nonunion, has become increasingly accepted by clinicians. *In vivo*, osteoblasts are affected by a variety of osteoblast growth factors, which can increase osteoblast activity and promote their proliferation and differentiation. Nonetheless, any kind of osteogenic growth factor can have effect on promoting bone formation alone. Therefore, BMP, TGF-ß, CBF-α1, VEGF, IGF, FGF and PDGF have all shown to be involved in the process of sensing and responding to the biological effects of ESW. Growth factors are involved in a variety of signal transduction pathways, which contribute to the crosstalk among cells in tissue regeneration and which can guide and inspire the clinicians to explore the potential use of ESWT in clinical practice.

## Author contributions

All authors listed have made a substantial, direct, and intellectual contribution to the work and approved it for publication.
